# Simultaneous Determination of Celecoxib, Erlotinib, and its Metabolite Desmethyl-Erlotinib (OSI-420) in Rat Plasma by Liquid chromatography/Tandem Mass Spectrometry with Positive/Negative Ion-Switching Electrospray Ionisation

**DOI:** 10.3797/scipharm.1205-09

**Published:** 2012-06-18

**Authors:** Satheeshmanikandan R. S. Thappali, Kanthikiran Varanasi, Sridhar Veeraraghavan, Rambabu Arla, Sandhya Chennupati, Madheswaran Rajamanickam, Swaroop Vakkalanka, Mukkanti Khagga

**Affiliations:** 1 Incozen Therapeutics Pvt. Ltd, Alexandria Knowledge Park, Shamirpet, Hyderabad, 500078, India.; 2 Centre for Pharmaceutical Sciences, JNT University, Kukatpally, Hyderabad, India.

**Keywords:** Celecoxib, Erlotinib, Desmethyl erlotinib, OSI-420, LC-MS/MS, Bioanalytical

## Abstract

A new method for the simultaneous determination of celecoxib, erlotinib, and its active metabolite desmethyl-erlotinib (OSI-420) in rat plasma, by liquid chromatography/tandem mass spectrometry with positive/negative ion-switching electrospray ionization mode, was developed and validated. Protein precipitation with methanol was selected as the method for preparing the samples. The analytes were separated on a reverse-phase C_18_ column (50mm×4.6mm i.d., 3μ) using methanol: 2 mM ammonium acetate buffer, and pH 4.0 as the mobile phase at a flow rate 0.8 mL/min. Sitagliptin and Efervirenz were used as the internal standards for quantification. The determination was carried out on a Theremo Finnigan Quantam ultra triple-quadrupole mass spectrometer, operated in selected reaction monitoring (SRM) mode using the following transitions monitored simultaneously: positive m/z 394.5→278.1 for erlotinib, m/z 380.3→278.1 for desmethyl erlotinib (OSI-420), and negative m/z −380.1→ −316.3 for celecoxib. The limits of quantification (LOQs) were 1.5 ng/mL for Celecoxib, erlotinib, and OSI-420. Within- and between-day accuracy and precision of the validated method were within the acceptable limits of < 15% at all concentrations. The quantitation method was successfully applied for the simultaneous estimation of celecoxib, erlotinib, and desmethyl erlotinib in a pharmacokinetic study in Wistar rats.

## Introduction

The epidermal growth factor receptor (EGFR) is recognized as an important molecular target in cancer therapy. Erlotinib (ERT) is an orally-active and potent inhibitor of the EGFR tyrosine kinase (TKI) used in lung cancer and several other cancers [1, 2]. Celecoxib (CCB) is a selective cyclooxygenase-2 (COX-2) inhibitor [3]. COX-2 is an inducible enzyme that is overexpressed in pancreatic cancer. Through the conversion of arachidonic acid to prostaglandin, the COX-2 enzyme modulates angiogenesis and metastasis. This currently ongoing clinical trial utilizes erlotinib and celecoxib in non-small-cell lung cancer/head and neck cancer [4, 5]. A fast, sensitive, and specific LC/MS/MS method for the simultaneous determination of ERT and its active metabolites in rat plasma, OSI-420 and CCB, is described. After administration of ERT and CCB, blood samples were periodically collected from male Wistar rats. The pharmacokinetic parameters of ERT, OSI-420, and CCB were calculated.

Literature reviews reveal that methods have been reported for the analysis of CCB, ERT, and OSI-420, and the HPLC and LC-MS/MS method for the determination of CCB, ERT, and OSI-420 in combination with other drugs was also reported [6–17]. To the best of our knowledge, no prior reports have described a LC–MS/MS-based method for the simultaneous determination of ERT, OSI-420 & CCB from plasma. Hence, we developed a reverse-phase HPLC method for the simultaneous estimation of ERT, OSI-420 & CCB on a C_18_ column using tandem mass spectroscopy detection, and validated the method before applying it in preclinical experiments. The current study describes a rapid, specific, and simple protein precipitation method using LC-MS/MS for the simultaneous determination of CCB and ERT, along with its major active metabolite OSI-420 in rat plasma, which is suitable for pharmacokinetic and drug-drug interaction studies. The method was validated using authentic pure standards. This method was successfully applied to the pharmaco-kinetic study of CCB with ERT in rats after oral administration of CCB and ERT.

## Materials and Methods

### Chemical and Reagents

Pure reference standards of celecoxib (CCB), Erlotinib (ERT), and Sitagliptin (SIT) were obtained from Sigma-Aldrich (Germany). Desmethyl erlotinib (OSI-420) was obtained from Selleckchem (USA). Efavirenz (EFV) was obtained from SBC (USA). Acetonitrile (HPLC grade), methanol (HPLC grade), and ammonium acetate (GR-grade) were procured from E Merck (India) Ltd., India. Formic acid was obtained from Sigma-Aldrich (Germany). Ultra pure water of 18 MΩ/cm was obtained from Millipore: Milli-Q purification system (USA).

### Stock solution, calibration standards and quality control samples

Standard stock solutions of CCB, ERT, OSI-420, SIT, and EFV were prepared in methanol with a final concentration of 1 mg/ml. These solutions were stored at 2–8°C until use. SIT and EFV stock solutions were diluted to achieve a final concentration of 1.2 μg/mL and 1.15 μg/mL, respectively, with the methanol. Analytical standards for CCB, ERT, and OSI-420 were prepared in acetonitrile: water (70:30, v/v) over a concentration range of 1.6 ng/mL to 1144.4 ng/mL, 1.8 ng/mL to 1289.2 ng/mL, and 1.5 ng/mL to 1177.2 ng/mL, respectively, by serial dilution, and the same concentration range for a calibration curve was prepared in the blank, normal rat plasma. Quality control (QC) samples at four different concentration levels 1.6, 3.9, 384.5, 915.6 ng/mL for CCB, 1.8, 4.4, 433.2, 1031.4 ng/mL for ERT, and 1.5, 3.8, 375.4, 893.8 ng/mL for OSI-420 as LLOQC (lower limit of quantitation QC, and low, medium, and high level of QC, respectively) were prepared in three sets, independent of the calibration standards. During analysis, low, medium, and high QC samples were placed after every sixth position of the unknown samples.

### Sample preparation

An aliquot of 50 μL of plasma was transferred to a 1 mL Eppendorf microcentrifuge tube, and 150 μL of internal standard solution (containing the final concentration of 1.2 μg/ml and 1.15 μg/ml, for SIT and EFV, in methanol) was added and the sample was vortex-mixed for 5 min. After centrifuging at 10000 rpm for 5 min at 4°C, 0.1 mL of the supernatant was collected and placed in the HPLC, with 10 μL injected onto the LC–MS/MS system.

### Chromatographic condition

A Shimadzu SIL – 20 AC HT (Shimadzu Corporation, JAPAN) consisted of a flow control valve and a vacuum degasser operated in isocratic mode to deliver the mobile phase at a flow rate of 0.8 ml/min. The chromatographic system consisted of a reverse-phase C_18_ column (50mm×4.6mm i.d., 3μ) (YMC^®^-PACK, JAPAN) and the mobile phase consisted of 80% v/v solvent A: methanol and 20% v/v solvent B: ammonium acetate buffer, 2mM (pH ~4.0 adjusted with 0.1% formic acid). The samples (10 μL) were injected onto the LC-MS/MS system through an auto injector. The auto sampler temperature was kept at 10°C and the column oven was maintained at 40°C.

### Mass spectrometric condition

Mass spectrometric detection was performed on Thermo Scientific - Finnigan TSQ Quantum Ultra tandem mass spectrometer equipped with a Heated Electron Spray Ionization (HESI) source (San Jose, CA, USA), and Selective Reaction Monitoring (SRM) mode was used for data acquisition with Xcalibur 1.2 software(Thermo-Scientific, San Jose, CA, USA). Peak integration and calibration were carried out by using LC Quan 2.5.2 software (Thermo-Scientific). MS and MS/MS conditions for pure standards of CCB, ERT, OSI-420, SIT, and EFV were optimized by continuous infusion at 5μl/min, using a built-in syringe pump. The transitions monitored were *m/z* −380.1 > −316.3, 394.5 > 278.1, 380.3 > 278.1, and 408.1 > 127.0, −313.8 > −243.9 for components CCB, ERT, OSI-420, SIT, and EFV, respectively. All analyses were carried out in a positive and negative ion HESI, set for positive polarity, spray voltage at 3.5 KV, and heated capillary temperature 150°C. Nitrogen sheath gas and auxiliary gas were set at 40 and 30 KPa. For negative polarity, the spray voltage was set at 3.0 KV, and the heated capillary temperature 300°C. The nitrogen sheath gas and auxiliary gas were set at 30 and 40 KPa, with the capillary offset at −35. The argon gas collision-induced dissociation was used with a pressure of 1.5 m Torr with the energy selected at 2100 eV. The total run time for an LC-MS/MS analysis was 5.0 min.

### Assay Validation

Specificity was assessed by analysis of six different samples of a blank matrix with and without spiking with CCB, ERT, OSI-420, and IS. Calibration curves were constructed from the working standard solutions of CCB, ERT, and OSI-420 at the concentration range 1.5–1150 ng/mL by plotting peak area ratio (y) of analyte(s) of the internal standard, versus analyte concentration (x). Linearity was assessed by weighted (1/x^2^) linear regression of calibration curves generated in triplicate on three consecutive days, using analyte internal standard peak area ratios. Quality control samples (around 1.5, 4, 400, and 900 ng/mL) were prepared to evaluate the accuracy, precision, recovery, stability, and matrix effect of the assay. Accuracy (expressed as percent nominal, SD) and between- and within-day precision (expressed as percent co-efficient of variation- %CV) were assessed by assay of six replicate QC samples on three different days.

The limit of quantification (LOQ) was defined as the lowest concentration in the calibration curve that can be determined with accuracy and precision of no more than 80–120%, ±20%, respectively. The limit of detection (LOD) was defined as a signal-to-noise ratio of 3:1. The extraction recovery for the analytes and IS were determined by assaying two sets of samples: plasma extracts spiked with analytes and IS after extraction (set 1), and plasma spiked with analytes and IS before extraction (set 2). CCB, ERT, and OSI-420 of each batch were prepared at levels of 4, 400, and 900 ng/mL. The percent extraction recoveries of CCB, ERT, OSI-420, and IS were calculated as the percent ratio of set 2 peak area to set 1 peak area.

Matrix effect was evaluated to verify whether potential ion suppression or enhancement, due to the co-elution matrix components, existed in the analysis. The peak areas of analytes and the internal standard from the spike-after protein precipitation samples were compared to those of the standard solutions in the mobile phase at the same concentrations. This experiment was carried out with blank plasma samples from six different rats at low and high QC concentrations of CCB, ERT, and OSI-420.

Potential sample carry-over was tested by analyzing the upper limit of quantitation (ULOQ, 1150 ng/mL) calibrator of CCB, ERT, and OSI-420 of respective samples, followed by blank samples.

Stability experiments were performed to evaluate the analyte stability in stock solutions and in plasma samples under different conditions. Stock solution stability was performed by comparing area response of the stability sample of the analyte with the area response of the sample prepared from fresh stock solutions. To meet the acceptance criteria, percent change should be within ±10% when compared to the fresh stock solution. Bench-top stability, long-term stability, freeze–thaw cycle stability, and autosampler stability were performed at 4 ng/mL and 900 ng/mL of QC levels using six replicates at each level. To meet the acceptance criteria, the nominal percentage should be within ±15% of its respective nominal concentrations.

The dilution integrity experiment was performed with an aim to validate the dilution test to be carried out on higher analyte concentrations (above ULOQ), which should be encountered during real sample analysis. A set of plasma samples was prepared containing CCB, ERT, & OSI-420 at a concentration of 4500, 5100, & 4500 ng/mL, respectively, and placed in a −70°C freezer overnight prior to analysis. After thawing, a certain aliquot was diluted 4 & 8 times with Wistar rat plasma and analyzed, respectively. The results of this experiment indicated that the dilution integrity of all the plasma samples was found to be less than 15% of their respective nominal concentrations.

### Application of method in pharmacokinetic study

Healthy male Wistar rats weighing 200 ± 30g were obtained from Mahaveera Enterprises, Hyderabad and housed at Incozen Therapeutics Pvt Ltd, Hyderabad in appropriate cages. They were maintained under standard laboratory conditions with a regular 12 h day–night cycle in a well-ventilated room with an average temperature of 24–27°C, and relative humidity of 40–60%. Standard pelleted laboratory chow diet (Provimi Animal Nutrition India Private Limited, Bengaluru, India) and water was given *ad libitum* to rats. All of the applicable national and international ethical guidelines for maintenance and experimental studies with Wistar rats were followed.

The method was successfully applied to generate the plasma concentration versus time profile of test drugs (CCB and ERT), as well as to detect its active metabolite (OSI-420) in plasma following simultaneous oral administration at 20 mg/kg dose of ERT, and 10 mg/kg dose of CCB in six male Wistar rats. Oral formulations were prepared in suspension form by triturating an accurately weighed amount of powdered compound in methyl cellulose solution (0.5%, w/v water) in a gravimetric dilution pattern. Oral doses of ERT and CCB (20, 10 mg/kg,) were administered using an oral gavage at 5 ml/kg volume in rats after an overnight fast (12 hr), and a fasting restriction was continued until 4 hr post-dose. The blood samples (0.15 ml) were collected from the retro-orbital sinus at predose, 10, 15, 30 min and 1, 2, 4, 6, 8, 12, and 24 hrs post-dose into K_2_-EDTA (dipotassium ethylene-diaminetetraacetic acid) tubes and were kept on an ice bath until further processing. These samples were separated for plasma by centrifugation at 4°C for 10 min at 3000 rpm and then stored at −70°C until further analysis. These samples were simultaneously estimated for the levels of CCB, ERT, and its active metabolite OSI-420.

Pharmacokinetic parameters, including the area under the concentration–time curve (AUC), maximum plasma concentration (C_max_), and time to reach the maximum concentration (T_max_), were estimated by means of a non-compartmental analysis using Phoenix WinNonlin (Pharsight Inc., USA, version 6.1). Statistical parameters like mean, standard deviation, and C.V were calculated by using MS-Excel 2007 (Microsoft^®^).

Incurred sample reanalysis (ISR) was performed to reconfirm the initial values, and to demonstrate that the assay was reproducible. In the study, ISR was performed on 18 plasma samples from six different rats at Tmax, and the second time the point covered the phase of elimination.

## Results and discussion

### Mass spectrometry

**Fig. 2:**
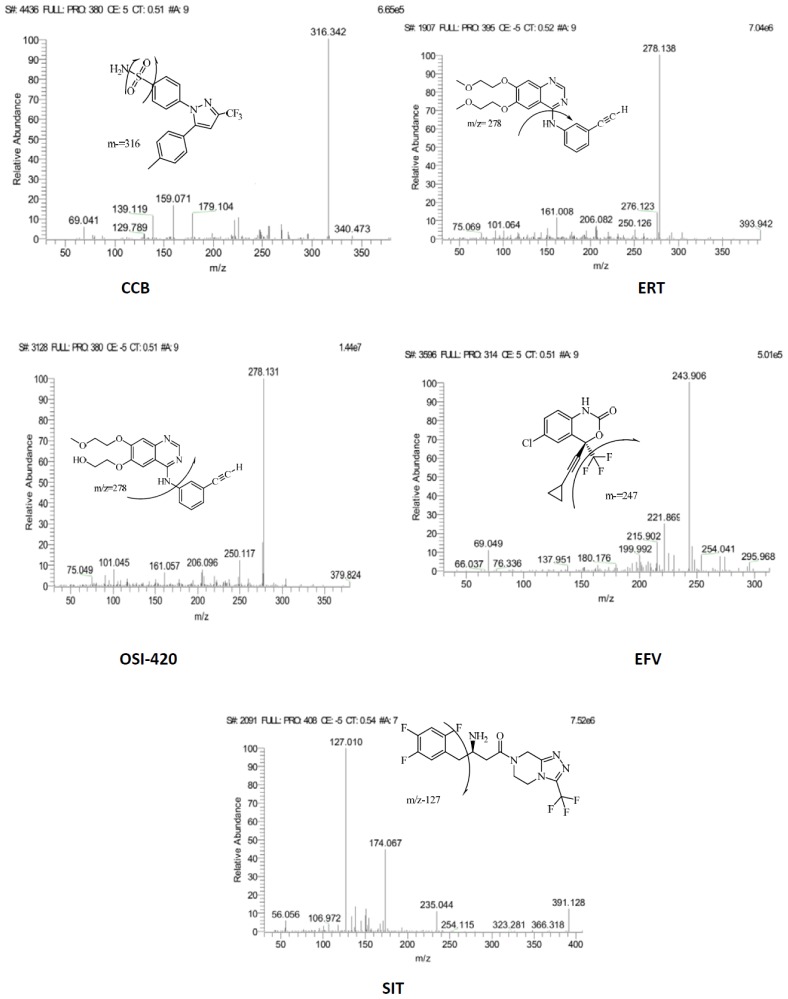
Fragmentation pattern and product ion spectra of CCB, ERT, OSI-420, EFV, and SIT.

In order to find most sensitive ionization mode for the components studied, ESI positive ion mode and ESI negative ion mode were tested with various combinations of mobile phases, i.e. acetonitrile and water/ammonium acetate buffer (2 mM)/formic acid (0.1%) in positive and negative ionization mode. It was observed that the signal intensity for [M + H]^+^ ions in ESI positive ion mode were 2–10-fold higher for ERT,OSI, and SIT in analyses using acetonitrile: ammonium acetate buffer (2 mM), versus experiments run with ESI negative ion mode. The protonated molecular ion of [M + H]^+^, *m/z* 446.4, 370.2, 368.3, and 491.4 amu were obtained for ERT, OSI-420, and SIT & [M – H]^−^, *m/z* −380.1 and −313 amu were obtained for CCB and EFV, respectively. No significant solvent adduct ions or fragment ions were observed in the full scan spectra of all the compounds. Thus, it was decided to utilize positive ion mode & negative ion switching mode for detection and quantitation, on which fragmentation gave prominent and stable product ions. The optimized tube-lens potentials for the protonated [M+H]^+^ of component ERT, OSI-420, and SIT were found to be 95, 67, and 122 eV, respectively, the negatively charged [M-H]– of component CCT, and EFV were found to be 110 and 76 eV, respectively. Product ions selected for final mass transition are given in Fig. 2.

### Liquid chromatography

**Fig. 3:**
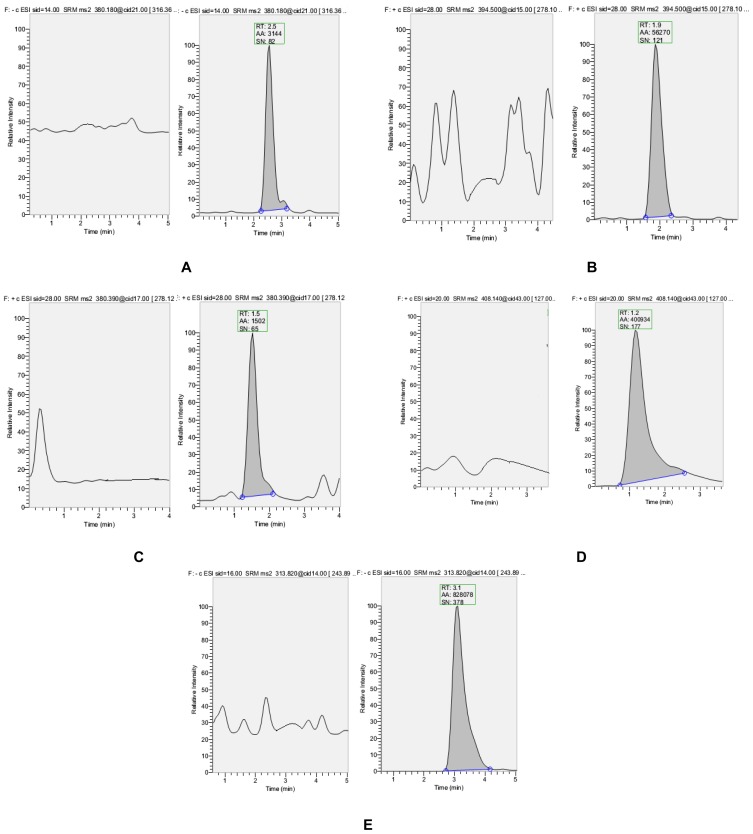
Representative chromatograms for (A) CCB, (B) ERT, C) OSI-420, (D) SIT, and (E)EFV in the extracted blank plasma and extracted LOQ rat plasma

Acetonitrile rather than methanol was chosen as the organic modifier because of the analytes’ better peak shape. Moderately high acidic ammonium acetate buffer 2 mM, pH~4, was required to achieve an acceptable peak width and shapes. A reverse-phase C_18_ column (50mm×4.6mm i.d., 3μ) (YMC-PACK®, JAPAN) with methanol: ammonium acetate buffer at flow rate of 0.8 mL/min in iscocratic mode, was applied in the final LC method. With the total analysis time of 5.0min, all components were eluted between in 1.2–4.2min. Representative chromatograms of blank and LOQ level are shown in Fig. 3.

### Optimization of LC–MS/MS condition

Final SRM transitions were selected on the basis of the signalto-noise ratio (S/N) with on-column injection analysis. The nitrogen sheath gas, auxiliary gas, argon gas collision- induced dissociation, ion spray voltage, and capillary temperature were set to 40 and 30 KPa, 1.5m Torr, 3500 eV, and 300°C, respectively. The transitions selected were *m/z* −380.1 > −316.3, 394.5 > 278.1, 380.3 > 278.1, and 408.1 > 127.0, −313.8 > −243.9 for components CCB, ERT, OSI-420, SIT, and EFV, respectively.

### Method validation

Accuracy, precision, selectivity, sensitivity, linearity, and stability were measured and used as the parameter to assess the assay performance. LC–MS/MS analysis of the blank plasma samples showed no interference with the quantification of components CCB, ERT, OSI-420, and internal standards. The specificity of the method was established with pooled and individual plasma samples from six different sources. The retention times of all the analytes and the IS showed less variability with a percent co-efficient of variance (% C.V) well within the acceptable limits of 5%.

#### Limit of detection (LOD) and quantification (LOQ)

Two criteria were used to define LOQ, i.e., (1) the analytical response at LOQ must be five times the baseline noise and (2) the analytical response at LOQ can be detected with sufficient accuracy (80–120%) and precision (20%). LOD is defined as the lowest concentration of the analyte at which the signal is larger than three times the baseline noise. The measured LOQ and LOD values were 20 and 5 arbitrary units for all three analytes. The LOQ was set at 1.5 ng/mL. These results easily met the requirements of quantifications of all analytes in plasma.

#### Linearity

The peak area ratios of analytes to IS in rat plasma were linear over the concentration range 1.6–1144.4 ng/mL for CCB and 1.8–1289.2 ng/mL for ERT and 1.5–1117.2 ng/mL for component OSI-420. The calibration model was selected based on the analysis of the data by linear regression with and without intercepts (y = mx + c and y = mx), and on weighting factors (1/x, 1/x^2^ and 1/log x). The best fit for the calibration curve could be achieved by a linear equation of y = mx + c and a 1/x^2^ weighting factor for all components. Mean slopes for CCB, ERT, and OSI420 were found to be 0.00026, 0.07124, and 0.00889, respectively. Mean intercepts for CCB, ERT, and OSI420 were found to be −0.00208, 0.01945, and −0.00522. The correlation coefficients (R^2^) for all components were above 0.99 over the concentration range used.

#### Precision and accuracy

The within-day precision (expressed by the coefficient of variation of replicate analyses) was estimated on the four quality control levels and the within-batch precision on the nine calibration standard levels. Table 1 shows the results obtained for the within-batch and between-batch precision for CCB, ERT, and OSI-420. The precision for all these analytes under investigation did not exceeded 15% at any of the concentrations studied and easily met the requirements of validation.

#### Recovery

The recoveries of CCB, ERT, and OSI-420 from plasma were estimated at their respective low, medium, and high QC levels. Plasma samples (in six replicates) containing all analytes at the QC concentration level were also spiked with their respective internal standards. The absolute recoveries ranged from 73.7 to 78.7%, 88.7 to 90.3%, and 92.2 to 94.4% for CCB, ERT, and OSI-420, respectively. The results are indicated in Table 2.

#### Stability

QC samples were subjected to short-term and long-term storage conditions (−70°C), freeze-thaw stability, autosampler stability, and bench-top stability studies. All stability studies were carried out at two concentration levels (low and high QC) in six replicates.

The bench-top stability was studied for low and high QC samples kept at room temperature (25°C) for six hours. Freeze-thaw stability of low and high QC samples was evaluated after three freeze-thaw cycles. The autosampler stability was studied for low and high QC samples which were stored at autosampler conditions at 10°C for 24 hours. The freezer-storage stability of the drug in plasma was determined by comparing the low and high QC samples stored for 30 days at −70°C. The results indicated that each analyte had an acceptable stability under those conditions, as shown in Table 3.

### Application to pharmacokinetic study

**Fig. 4:**
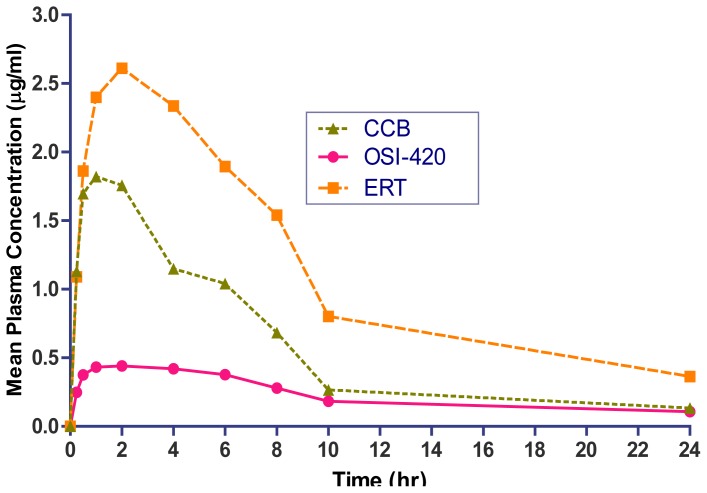
Mean plasma concentration vs. time after single-dose oral administration of CCB and ERT in six Wistar rats.

The method described above was successfully applied to a PK drug-drug interaction study, in which plasma concentration of pure markers was determined for up to 24 h after simultaneous oral administration at 20 mg/kg dose of ERT, and 10 mg/kg dose of CCB in male Wistar rats. The plasma concentration-time profiles of CCB, ERT, & its active metabolite OSI-420 are shown in Fig. 4, and could be traceable up to 24 h, 12h, and 12h, respectively. The pharmacokinetic parameters of CCB, ERT, and OSI-420 are presented in Table 4. During ISR, it was observed that all of the samples were within ±20% of the initial concentration values, further demonstrating that this method is capable of producing reproducible results over time.

## Conclusion

An LC–MS/MS bioanalytical method for the simultaneous determination of three analytes, CCB, ERT, and OSI-420, was developed and validated in rat plasma. The method was good enough to detect the low concentration of 1.5 ng/mL for all analytes in 50 μL rat plasma, and can further be improved by increasing the plasma volume. Analyte recoveries from spiked control samples were >73%, using a convenient and fast protein precipitation method. Intra- and inter-day accuracy and precision of the validated method were within the acceptable limits of <15% and 85 to 115%, respectively, at low and at <10% of other concentrations. This method was successfully applied to generate a stability profile as well as a PK evaluation of the simultaneous administration of CCB and ERT in rats following oral administration.

## Figures and Tables

**Fig. 1:**
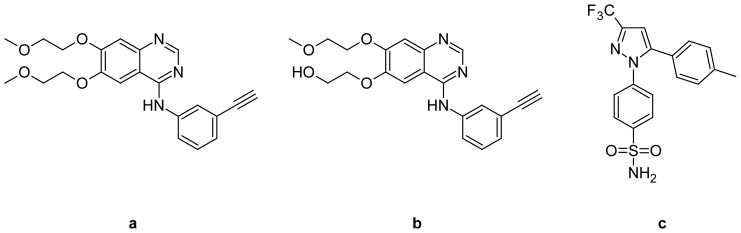
Structures of (a) Erlotinib, (b) Desmethly Erlotinib (OSI-420), and (c) Celecoxib

**Tab. 1:** Summary of precision and accuracy from QC samples in wistar rat plasma

Drug	Spiked concentr. (ng/mL)	Within batch (n=6)			Between batch (n=3)		

		Measured concentration (ng/ml) (mean ± SD)	% Accur.	% C.V	Measured concentration (ng/ml) (mean ± SD)	% Accur.	% C.V
CCB	1.6	1.64 ± 0.125	102.2	7.6	1.62 ± 0.139	101.0	8.6
	3.9	3.91 ± 0.263	100.2	6.7	3.95 ± 0.271	101.3	6.9
	384.5	405.07 ± 21.351	105.3	5.3	399.93 ± 20.331	104.0	5.1
	915.5	916.14 ± 56.283	100.1	6.1	921.38 ± 61.516	100.6	6.7

ERT	1.8	1.81 ± 0.179	100.3	9.9	1.85 ± 0.151	102.9	8.2
	4.4	4.29 ± 0.285	97.4	6.7	4.45 ± 0.394	101.1	8.9
	433.2	403.37 ± 31.347	93.1	7.8	432.34 ± 41.379	99.8	9.6
	1031.4	1058.52 ± 44.444	102.6	4.2	1010.71 ± 54.712	98.0	5.4
	1.5	1.48 ± 0.149	96.2	10.0	1.5 ± 0.138	97.5	9.2

OSI-420	3.8	3.84 ± 0.308	100.0	8.0	4.02 ± 0.231	104.7	5.7
	375.4	387.43 ± 17.18	103.2	4.4	389.71 ± 17.606	103.8	4.5
	893.8	930.88 ± 26.121	104.1	2.8	917.36 ± 41.916	102.6	4.6

**Tab. 2:** Extraction recovery in rat plasma (n=6)

Drug	Concentration (ng/ml)	Recovery (%)	% C.V
CCB	3.9	73.7	9.3
	384.5	72.7	9.4
	915.5	78.7	8.5
ERT	4.4	88.7	7.4
	433.2	90.3	6.5
	1031.4	86.3	6.4
OSI-420	3.8	93.3	3.4
	375.4	94.4	2.3
	893.8	92.2	3.3

**Tab. 3:** Stability of analytes in rat plasma (n=6)

Drug	Nominal concentration (ng/ml)	Sample condition							

		Bench top stability^a^		Autosampler stability^b^		Freeze-thaw stability^c^		30 days storage stability^d^	

		% Accur.	% CV	% Accur.	% CV	% Accur.	% CV	% Accur.	% CV
ERT	4.4	90.2	7.4	101	5.5	106.3	4.5	100.6	7.5
	1031.4	98.6	6.4	100.5	6.7	100.6	9.2	112.7	5.5

OSI-420	3.8	101.7	9.4	107.9	5.4	112.8	4.4	94	8.4
	893.8	103.6	4.3	103.2	9.7	109.7	6.5	93.2	6.6

CCB	3.9	96.8	7.2	99.1	2.3	97.5	4.4	105	4.4
	915.5	99.2	6.6	98.6	3.5	100	4.6	95.1	7.6

a Exposed at ambient temperature (25°C) for 6h;

b Kept at autosampler temperature (10°C) for 24h;

c After three freeze-thaw cycles;

d Stored at −70°C.

**Tab. 4:** Pharmacokinetic parameters (Mean ± S.D.) after single-dose oral administration of CCB and ERT simultaneously in Wistar rats

Parameters	Units	CCB	ERT	OSI-420 (metabolite)
C _max_	μg/ml	1.91 ± 0.2	2.72 ± 0.23	0.48 ± 0.07
AUC _0–24_	μg.h/ml	13.72 ± 0.67	27.2 ± 3.12	5.57 ± 0.54
AUC _0–inf_	μg.h/ml	14.94 ± 0.92	31.78 ± 6.44	7.48 ± 1.28
t _1/2_	h	6.19 ± 0.9	7.94 ± 2.15	11.96 ± 2.01
T _max_	h	1.08 ± 0.49	2.17 ± 0.98	2 ± 1.1
K _el_	h-1	0.11 ± 0.02	0.09 ± 0.02	0.06 ± 0.01
